# Melanin-Concentrating Hormone (MCH): Role in REM Sleep and Depression

**DOI:** 10.3389/fnins.2015.00475

**Published:** 2015-12-17

**Authors:** Pablo Torterolo, Cecilia Scorza, Patricia Lagos, Jessika Urbanavicius, Luciana Benedetto, Claudia Pascovich, Ximena López-Hill, Michael H. Chase, Jaime M. Monti

**Affiliations:** ^1^Department of Physiology, School of Medicine, Universidad de la RepúblicaMontevideo, Uruguay; ^2^Department of Experimental Neuropharmacology, Instituto de Investigaciones Biológicas Clemente EstableMontevideo, Uruguay; ^3^WebSciences International and University of California, Los Angeles School of MedicineLos Angeles, CA, USA; ^4^Department of Pharmacology and Therapeutics, School of Medicine, Hospital de Clínicas, Universidad de la RepúblicaMontevideo, Uruguay

**Keywords:** peptides, hypothalamus, serotonin, raphe, mood, paradoxical sleep, MCH

## Abstract

The melanin-concentrating hormone (MCH) is a peptidergic neuromodulator synthesized by neurons of the lateral sector of the posterior hypothalamus and zona incerta. MCHergic neurons project throughout the central nervous system, including areas such as the dorsal (DR) and median (MR) raphe nuclei, which are involved in the control of sleep and mood. Major Depression (MD) is a prevalent psychiatric disease diagnosed on the basis of symptomatic criteria such as sadness or melancholia, guilt, irritability, and anhedonia. A short REM sleep latency (i.e., the interval between sleep onset and the first REM sleep period), as well as an increase in the duration of REM sleep and the density of rapid-eye movements during this state, are considered important biological markers of depression. The fact that the greatest firing rate of MCHergic neurons occurs during REM sleep and that optogenetic stimulation of these neurons induces sleep, tends to indicate that MCH plays a critical role in the generation and maintenance of sleep, especially REM sleep. In addition, the acute microinjection of MCH into the DR promotes REM sleep, while immunoneutralization of this peptide within the DR decreases the time spent in this state. Moreover, microinjections of MCH into either the DR or MR promote a depressive-like behavior. In the DR, this effect is prevented by the systemic administration of antidepressant drugs (either fluoxetine or nortriptyline) and blocked by the intra-DR microinjection of a specific MCH receptor antagonist. Using electrophysiological and microdialysis techniques we demonstrated also that MCH decreases the activity of serotonergic DR neurons. Therefore, there are substantive experimental data suggesting that the MCHergic system plays a role in the control of REM sleep and, in addition, in the pathophysiology of depression. Consequently, in the present report, we summarize and evaluate the current data and hypotheses related to the role of MCH in REM sleep and MD.

## Introduction

The search for new pharmacological strategies to treat psychiatric disorders is a “hot topic” in neuroscience. There is also a large body of evidence suggesting that neuropeptides play a critical role in these pathologies (Kormos and Gaszner, 2013). In addition, neuropeptides are also involved in the control of wakefulness and sleep (Richter et al., 2014). A robust example of the results of translational neuroscience research was the discovery of the neuropeptides hypocretins 1 and 2 (also called orexins); the degeneration of hypocretins-containing neurons produces the sleep pathology called narcolepsy (Mignot, 2011). Hypocretins also play a role in mood disorders (Nollet and Leman, 2013).

Research on the neuropeptide melanin-concentrating hormone (MCH) is currently focused on the study of physiological and translational possibilities of the neuropeptide. In the present report, we review the role of the MCHergic system in the control of REM sleep and the pathophysiology of major depression (MD).

## Pathophisiology of depression: role of the serotonergic system

The Diagnostic and Statistical Manual of Mental Disorders (DSM-5) lists several depressive disorders. The common features that characterize them are the presence of sadness, empty or irritable moods, which are accompanied by somatic and cognitive changes that significantly affect an individual's capacity to function. Major Depressive Disorder or MD is a distinctive condition that is diagnosed based on symptomatic criteria such as sadness or melancholia, guilt, irritability and anhedonia. It is accompanied by several symptoms including insomnia or hypersomnia, fatigue, alterations in body weight, thought and concentration impairments as well as recurrent suicidal thoughts (Fava and Kendler, 2000; American-Psychiatric-Association, 2013). MD is one of the most common psychiatric diseases with a prevalence of 5–12% in men and 10–25% in women, and it is considered to be a principal cause of disability, outnumbered only by cardiovascular diseases (Boyd and Weissman, 1981; Murray and Lopez, 1996, 1997a,b). In addition, a great deal of attention has been paid to MD due to its association with suicide; 15–20% of depressive patients end their lives by suicide (Miret et al., 2013). Affective disorders are also particularly disabling and among the most important contributors to the total burden of disease (Miret et al., 2013).

Several mechanisms have been involved in the neurobiology of depression, ranging from synaptic plasticity to epigenetic, and from postnatal neurogenesis to immunological processes (recently reviewed by Palazidou, 2012; Saveanu and Nemeroff, 2012; Palagini et al., 2013; Willner et al., 2013; Ménard et al., 2015). In the present report, we review the role of MCH in depression with an emphasis on its interaction with the serotonergic system. However, it is important to note that in addition to serotonin, other neurotransmitters such as dopamine, noradrenaline, and glutamate have been involved in the pathophysiology of depression; in fact ketamine, a N-methyl-d-aspartate (NMDA) glutamate receptor antagonist can alleviate depressive symptoms in patients within hours of administration (Saveanu and Nemeroff, 2012; Dutta et al., 2015).

The serotonergic system comprises one of the most widely distributed neurochemical systems in the central nervous system (CNS). The majority of the somata of serotonergic neurons are located within the dorsal raphe nucleus (DR); another important group of serotonergic neurons is located in the median raphe nucleus (MR). Due to its projections throughout the CNS, serotonergic neurons are capable of playing an important role in the regulation of emotional states and in several functions including motor activity and the control of sleep and wakefulness (Monti, 2010a,b; Olivier, 2015).

Numerous studies have shown that the serotonergic system is involved in the pathophysiology of MD. Low levels of serotonin and/or its principal metabolite (5- hydroxy-indol-acetic acid) have been found in the urine and cerebrospinal fluid (CSF) of MD patients (Praag, 1977; van Praag and de Haan, 1979; Young, 1993). In addition, the number of attempted suicides by MD patients and its lethality are correlated with a reduced CSF concentrations of serotonin (Roy et al., 1989; Träskman-Bendz et al., 1992; Nordström et al., 1994; Mann et al., 2001; Kalia, 2005; Berton and Nestler, 2006). Furthermore, abnormalities in serotonergic receptors, serotonin reuptake proteins and other alterations in serotonergic neurotransmission have been related to the susceptibility to commit suicide (Arango et al., 1995; Mann, 1998; Courtet et al., 2005; Hamet and Tremblay, 2005; Bondy et al., 2006). Depressive-suicide patients show significant differences in serotonergic markers in the DR compared to control individuals. Therefore, it has been suggested that disruption of the functioning of serotonergic neurons in the DR underlies MD (Underwood et al., 1999; Arango et al., 2001, 2002; Boldrini et al., 2005; Bach-Mizrachi et al., 2006).

The serotonergic system is also involved in the mechanisms of action of antidepressant drugs. The selective serotonin reuptake inhibitors (SSRI) such as fluoxetine or escitalopram, produce its therapeutic action upon the enhancement of central serotonergic neurotransmission (Keller et al., 1992; Holtzheimer and Nemeroff, 2006). A problem related with antidepressant drugs treatment is the delayed onset of the therapeutic effects, despite the fact that these drugs cause an immediate increase in extracellular levels of monoamines. This fact suggests that the acute biochemical effect does not directly determine the therapeutic effect, which is likely produced by slower neurobiological modulations, such as the desensitization of serotonergic receptors, the modulation of intracellular pathways, gene expression of growth factors such as brain-derived neurotrophic factor (BDNF) and the regulation of postnatal neurogenesis (Palazidou, 2012; Saveanu and Nemeroff, 2012; Willner et al., 2013). In spite of the previous statements, it is important to take into account that a meta-analyses study showed that the placebo effect for antidepressant treatment is exceptionally large, and antidepressant medications have reported only modest benefits over placebo treatment (Kirsch et al., 2008; Kirsch, 2014).

## REM sleep

Sleep remains one of the great neurobiological mysteries. Humans spend one third of their life sleeping, without awareness of the outside world. However, during dreams, there is bizarre cognitive activity that is disconnected from reality and is ruled by internal stimuli (Pace-Schott, 2005).

In mammals, sleep is comprised of two different behavioral states: slow wave sleep, also called non-REM (NREM) sleep and REM (rapid eyes movements) sleep (Carskadon and Dement, 2005; Brown et al., 2012). Polysomnography is the basic tool to study behavioral states; it consists in the simultaneous recording of the electroencephalogram (EEG), electromyogram (EMG), and eye movements (electrooculogram). During wakefulness, there is an optimal interaction with the environment that enables to carry out different behaviors that optimize survival. An EEG consisting of high frequency rhythms and low amplitude waves characterizes wakefulness. In normal adults, NREM sleep occurs at sleep onset. During this period, there is a marked decrease in the interaction with the environment, the adoption of a suitable position to conserve heat, an increase in the threshold of reaction to external stimuli and a decrease in muscle activity and tone. During NREM sleep, the EEG exhibits low frequency (0.5–4 Hz), high amplitude waves and “sleep spindles.” NREM sleep is accompanied by a tonic increase in parasympathetic activity, which results in a decrease in visceral activity (Parmeggiani, 1994). In the deepest stage of NREM sleep, cognitive processes (dreams) are absent or minimal (Pace-Schott, 2005).

During a typical night's sleep in a young adult, REM sleep, which occurs with an ultradian rhythm of approximately 90 min, is always preceded by NREM sleep. During REM sleep the EEG is similar to wakefulness (consequently, this state is also called paradoxical sleep). REM sleep is also characterized by the absence of muscle activity (muscle atonia), rapid eyes-movements, ponto-geniculo-occipital (PGO) waves, theta waves in the hippocampus electrogram, and phasic changes in autonomic activity (Carskadon and Dement, 2005; Siegel, 2011). The arousal threshold for sound stimulation in humans during tonic REM sleep is similar than NREM sleep stage 2, and increases during phasic REM sleep (when rapid eyes movements are present) to the same level as NREM sleep stage 4 (Ermis et al., 2010). Dreams are present mostly during REM sleep (Pace-Schott, 2005).

It is well established that the activating system, which is a neuronal network centered in the mesopontine reticular formation, the postero-lateral hypothalamus and basal forebrain, is critical for generating and maintaining wakefulness (Torterolo and Vanini, 2010). The activating system comprises various neurochemical groups of neurons including glutamatergic, serotonergic, dopaminergic, noradrenergic, histaminergic, cholinergic and hypocretinergic (Torterolo and Vanini, 2010; Monti, 2013). On the other hand, the preoptic area is essential for the generation of NREM sleep, while the thalamus is responsible for the generation of slow waves and sleep spindles (Steriade et al., 1993; Torterolo et al., 2009a; Torterolo and Vanini, 2010; Benedetto et al., 2012).

The neuronal network that is “necessary and sufficient” for the generation of REM sleep is located in the mesopontine reticular formation (Siegel, 2011). Within this region, cholinergic neurons of the latero-dorsal and pedunculo-pontine tegmental nucleus (LDT-PPT) as well as glutamatergic neurons of the nucleus pontis oralis (NPO, that is considered the executive area for REM sleep generation) are active during REM sleep (REM “on,” or wake and REM “on” neurons), whereas noradrenergic neurons of the locus coeruleus (LC) as well as serotonergic neurons of the DR and MR suppress their firing (REM “off” neurons; Monti, 2010b; Siegel, 2011; Brown et al., 2012; Chase, 2013a; Boucetta et al., 2014). Neurons of the ventrolateral periaqueductal gray may also play a role in REM sleep generation (Vanini et al., 2007). Mutual interactive models that include REM “on” and REM “off” neurons have been presented in order to explain the generation of REM sleep (Lu et al., 2006; Luppi et al., 2007; Brown et al., 2012). The mesopontine REM sleep-generating neuronal network is strongly modulated by forebrain sites. This region receives important MCHergic and hypocretinergic projections from the hypothalamus (Torterolo et al., 2009b, 2013).

## Depression and REM sleep

A decrease in the latency to the first episode of REM sleep is a trait of MD, and is considered to be one of the most robust and specific biological markers of this condition (Adrien, 2002; Palagini et al., 2013). Furthermore, MD patients have an increase in the total time spent in REM sleep, in the length of the first episode of REM sleep and in the density of rapid eye movements during this state. The fact that most antidepressant drugs decrease or eliminate REM sleep and that selective REM sleep or total sleep deprivation are effective in the treatment of MD, highlights the relationship between MD and REM sleep (Adrien, 2002; Benca, 2005; Palagini et al., 2013). In relation with preceding concepts, serotonergic neuronal activity in the DR is reduced in animal models of depression, while the firing rate of such DR neurons is exacerbated during total sleep deprivation (which appears to be correlated with its antidepressant effect; Yavari et al., 1993; Gardner et al., 1997).

One of the most widely used preclinical paradigm for assessing antidepressant activity is the forced swimming test (FST) (Porsolt et al., 1977; Porsolt, 2000). When rodents are placed in an inescapable container of water, they swim or climb more following the systemic administration of antidepressant. Thus, these agents prevent an immobility state called “behavioral despair.” Passive (immobility) or active (swimming and/or climbing) responses should be independent from alterations in locomotive activity that the drug may also induce. Strikingly, 24-h of sleep deprivation in rats results in a decrease in the immobility time, which is consistent with an antidepressant effect (Lopez-Rodriguez et al., 2004). Sleep deprivation also enhances the effect of antidepressant drugs treatment (van Luijtelaar and Coenen, 1985). The antidepressant effect of sleep deprivation has been linked to an increase in serotonergic activity (Lopez-Rodriguez et al., 2004).

In conclusion, it has been proposed that patients with MD have an increased “pressure” to generate REM sleep; in other words, the generation of REM sleep is abnormally promoted or facilitated in these patients. The relationship between REM sleep and MD depends, at least in part, on the activity of serotonergic neurons of the DR. These neurons are involved both in the generation of REM sleep and in the pathophysiology of MD (Adrien, 2002; Palagini et al., 2013).

## Role of the serotonergic system in REM sleep

Serotonergic neurons of the DR have a slow and regular firing during wakefulness, there is a decrease in their activity during NREM sleep, and an almost complete inactivation during REM sleep (“REM-off” neurons; Monti, 2010b). A decrease in the release of serotonin in brain areas during REM sleep correlates with the electrophysiological data of DR neuronal activity (Portas and McCarley, 1994). GABAergic neurons of the DR are involved in the inhibition of serotonergic neurons during REM sleep, and the consequent promotion of this behavioral state (Nitz and Siegel, 1997; Torterolo et al., 2000; Monti, 2010a). On the other hand, experimental activation of DR serotonergic neurons prevents the generation of REM sleep (Monti, 2010a). Hence, the generation of REM sleep depends on the inactivation of the serotonergic neurons; these neurons are considered “permissive” for the generation of this behavioral state.

## The melanin-concentrating hormone

**Figure 1:**
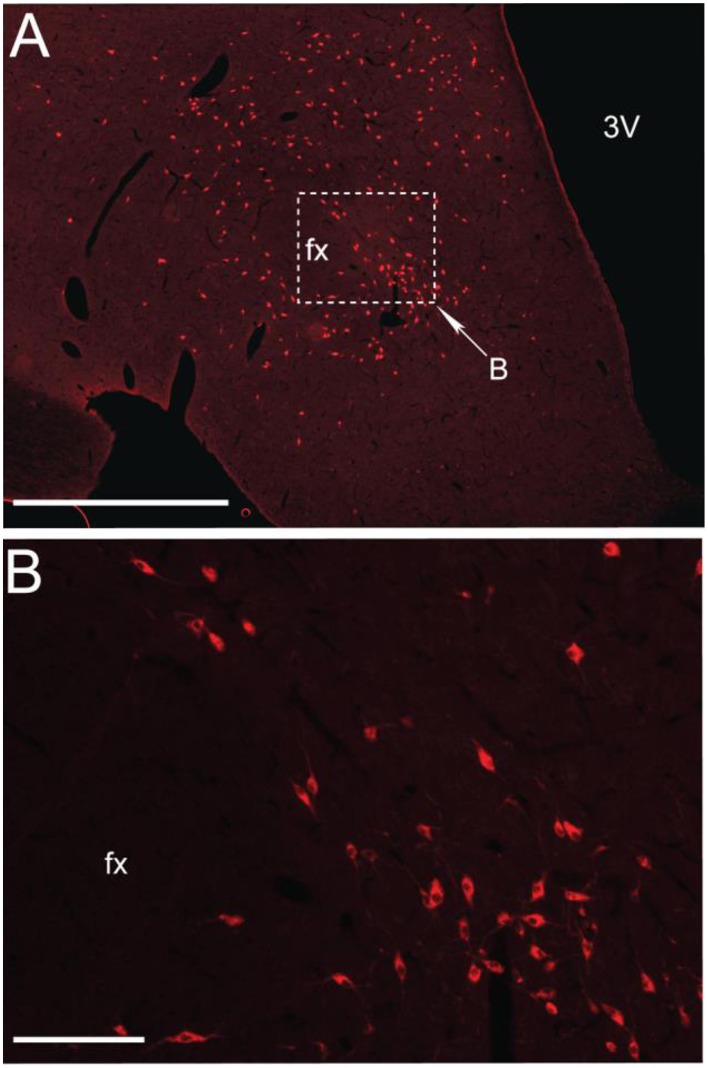
**MCHergic neurons are located in the hypothalamus**. **(A)** Low magnification photomicrographs that exhibit MCHergic neurons at the tuberal level of the hypothalamus of the cat. **(B)** The inset in **(A)** is shown at higher magnification. This photomicrograph shows MCHergic neurons of the perifornical region. The photomicrographs were taken from 20 μm -thick sections that were processed for immunofluorescence. Fx, fornix; 3 V, third ventricle. Calibration bars: **(A)** 1 mm; **(B)** 100 μm. Original microphotographs taken from the data set of Torterolo et al. (2006).

MCH is a 19-aminoacids cyclic peptide, which was initially characterized as a circulating factor that mediated color changes in teleost fishes (Torterolo et al., 2011; Macneil, 2013; Monti et al., 2013). MCH was subsequently identified as a neuromodulator in mammals, including humans (ibid.). MCH is synthesized in neurons whose somata are located in the lateral sector of the posterior hypothalamus, dorsomedial hypothalamus and zona incerta; the location of the MCHergic neurons is shown in a coronal section of the hypothalamus of the cat in Figure 1. These neurons project to different regions of the CNS (Bittencourt et al., 1992; Torterolo et al., 2006, 2009b). A small number of MCHergic neurons have been also identified in the olfactory tubercle and the pontine reticular formation (Bittencourt et al., 1992). In addition, MCHergic neurons are present in the medial preoptic area of lactating rats and in the latero-dorsal tegmental nucleus of female rats (Rondini et al., 2007, 2010). MCH is also present in the gastrointestinal tract and pancreas (Pissios et al., 2007; Kokkotou et al., 2008).

The biological functions of MCH are mediated by two G-protein coupled receptors known as MCHR-1 and MCHR-2. Of note, the MCHR-2 gene is a pseudo-gene in rodents but it is functional in carnivores and primates including humans. It has been determined that MCHR-1 activates Gi and Gq proteins and inhibits Ca^2+^ currents. MCH has mainly an inhibitory role, both at the presynaptic level where it decreases the release of GABA and glutamate, and at the post-synaptic level (Gao, 2009; Macneil, 2013).

### MCH regulates the energy homeostasis

The MCHergic system was traditionally related to the control of energy homeostasis; i.e., feeding and metabolic activity (reviewed by Macneil, 2013). In this regard, chronic infusion of a synthetic MCHR-1 receptor agonist induces obesity in mice, which is accompanied by hyperphagia, a reduction in body temperature and stimulation of lipogenic activity in the liver as well as white adipose tissue (ibid.). At the same time, genetically-modified animals with over-expression of MCH are obese, whereas animals lacking MCH are hypophagic and lean. These data suggest that by increasing food intake and promoting anabolism, MCH promotes the conservation of body energy.

### The MCHergic system promotes sleep

Conservation of energy is one of the main functions of sleep (Siegel, 2005). Since MCHergic neurons are critical in the control of energy homeostasis, it is expected to be involved in sleep regulation. MCH, via regulation of the activating and somnogenic systems, promotes sleep, especially REM sleep (reviewed by Torterolo et al., 2011; Monti et al., 2013; Konadhode et al., 2015).

The arguments that support MCH as a sleep promoter are described below. The main experimental results are summarized in Table 1.

**Table 1:** **MCH and sleep**.

**Site**	**Main effect**	**References**
**MCH MICROINJECTION**		
Intracerebroventricular (rat)	Increases REM sleep. Moderate increase in NREM sleep	Verret et al., 2003
Dorsal raphe (rat)	Increases REM sleep. Moderate increase in NREM sleep	Lagos et al., 2009
Locus coeruleus (rat)	Increases REM sleep	Monti et al., 2015
Nucleus pontis oralis (cat)	Increases REM sleep	Torterolo et al., 2009b
Basal forebrain (rat)	Decreases wakefulness. Increases REM sleep in the first 2-h of the recordings	Lagos et al., 2012
Ventro-lateral preoptic nucleus (VLPO) (rat)	Increases NREM sleep	Benedetto et al., 2013
**MCHR-1 ANTAGONIST MICROINJECTION**		
Systemic (rat)	Decreases REM and NREM sleep. Increases wakefulness	Ahnaou et al., 2008
**Type**	**Main effect**	**References**
**KNOCK-OUT ANIMALS**		
Prepro-MCH (mice)	Sleep less in basal condition. Decreases REM sleep during fasting more than wild-type controls	Willie et al., 2008
MCHR-1 (mice)	Hypersomniac phenotype both in basal conditions and after total sleep deprivation	Adamantidis et al., 2008
MCHR-1 (mice)	Increases wakefulness and reduces NREM sleep. Restraint stress reduced both NREM and REM sleep more than wild-type controls	Ahnaou et al., 2011
**Optogenetic**	**Main effect**	**References**
**STRATEGY**		
Stimulation of MCHergic neurons (mice)	Increases NREM and REM sleep	Konadhode et al., 2013
Stimulation of MCHergic neurons at the onset of REM sleep (mice)	Increases REM sleep duration	Jego et al., 2013
Stimulation of MCHergic neurons (mice)	Induces transitions from NREM to REM sleep and increases REM sleep time	Tsunematsu et al., 2014
Inhibition of MCHergic neurons at the onset of REM sleep (mice)	Reduces the frequency and amplitude of hippocampal theta rhythm	Jego et al., 2013
Inhibition of MCHergic neurons (mice)	No effect	Tsunematsu et al., 2014
**Electrophysiology**	**Main effect**	**Reference**
***IN VIVO*** **RECORDINGS**		
Identified MCHergic neurons (rat)	Firing rate: REM >NREM sleep>W	Hassani et al., 2009
***In vivo*** **Microdialysis**	**Main effect**	**Reference**
**SITE**		
Amygdala (human)	MCH release increases during NREM sleep onset	Blouin et al., 2013

### MCHergic neurons project to the activating and limbic systems

Classical studies have linked the postero-lateral hypothalamus, where MCHergic neurons are located, with the control of sleep and wakefulness (Torterolo and Vanini, 2010). These neurons have a close anatomical relationship with hypocretin-containing neurons, whose somata are also located within the postero-lateral hypothalamus and project to comparable brain sites (Torterolo et al., 2006, 2009b, 2013; Torterolo and Chase, 2014). Hypocretinergic neurons are considered part of the activating system, and are essential for the maintenance of wakefulness (Torterolo and Vanini, 2003; Chase, 2013b; Torterolo and Chase, 2014). Hence, it is likely that MCHergic and hypocretinergic neurons interact, in a complementary mode, in order to regulate wakefulness and sleep (Torterolo and Chase, 2014).

**Figure 2:**
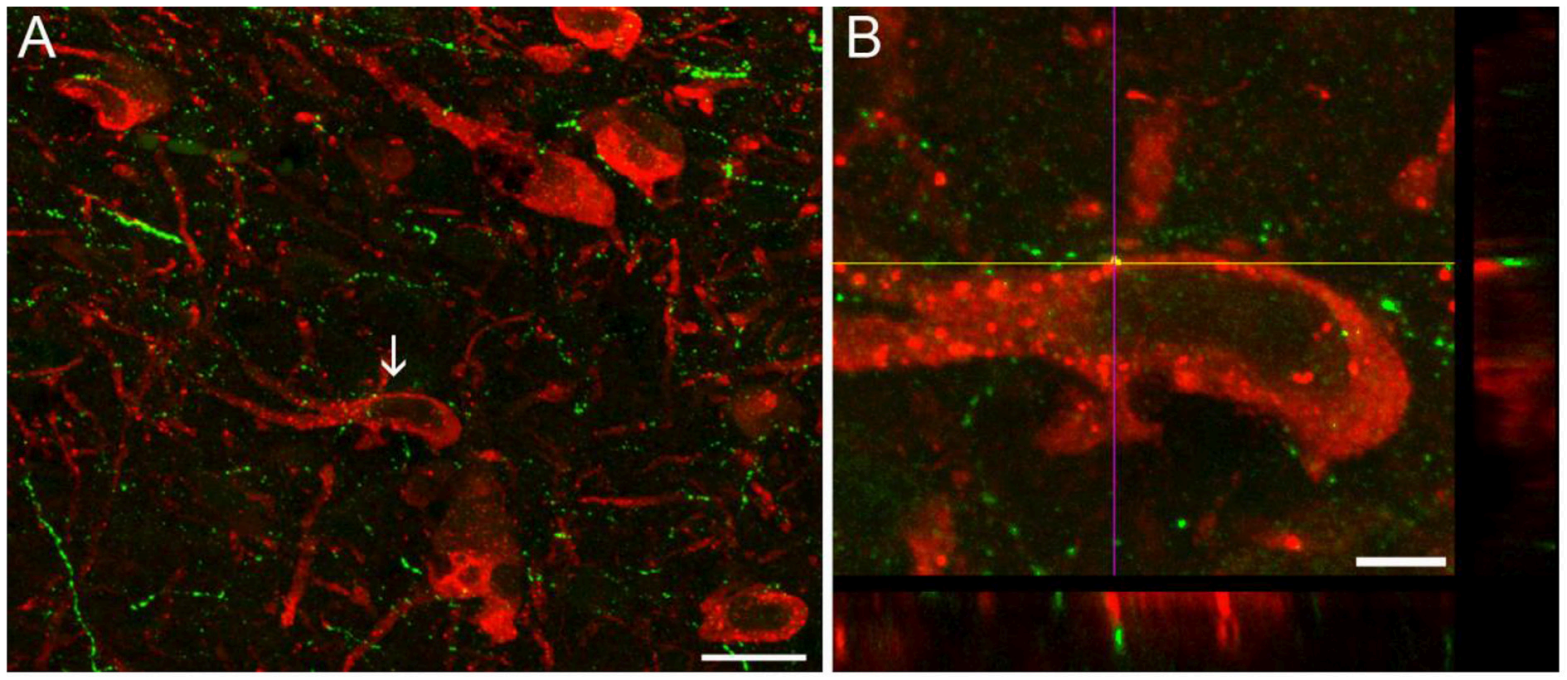
**Images of dual-immunostaining for 5-HT and MCH in the dorsal raphe nucleus**. Coronal sections (30 μm thickness) were double-labeled to visualize 5-HT- (red) and MCH−immunoreactivity (green). **(A)** MCH+ fibers were observed as small beaded processes around 5−HT+ neurons, intermingled with 5−HT+ neurons located in the mid-rostral level of the DR of the rat, according to Paxinos and Watson (2005). Arrow indicates the neuron in **(B)** that is shown at a high magnification (100X). **(B)** Orthogonal views (xz and yz) reveal apposition between MCH+ fibers and 5-HTergic soma. Image in **(B)** is comprised of 45 optical sections of 0.1 μm. Scale bars **(A)** 20 μm; **(B)** 5 μm. Original microphotographs taken from the data set of Urbanavicius et al. (2015b).

MCHergic neurons send dense projections to activating and somnogenic regions (Monti et al., 2013). Using retrograde tracers, we have characterized the MCHergic neuronal projections to the NPO (Torterolo et al., 2009b). There is also a high density of MCHergic fibers in activating regions such as DR and LC (Torterolo et al., 2008; Lagos et al., 2011b; Yoon and Lee, 2013). MCHergic fibers in the DR and their anatomical relation with serotonergic neurons are shown in Figure 2. Regions of the limbic system involved in the control of emotional states including amygdala, nucleus accumbens, septum and hippocampus also receive MCHergic fibers and express MCH receptors (Bittencourt et al., 1992; Hervieu et al., 2000; Chee et al., 2013).

We have also documented that tanycytes in the DR of the cat exhibit immunoreactivity to MCH (Torterolo et al., 2008). Tanycytes are specialized cells whose somata lies in the ependymal or sub-ependymal region that present long basal processes that projected deeply into the subventricular parenchyma. These cells absorb substances present in the CSF and transport them to the neuronal parenchyma (Rodríguez et al., 2005). These data, together with the presence of MCH in the CSF of the rat, sheep and humans (Peyron et al., 2011; Ungerfeld et al., 2011; Pelluru et al., 2013), suggest that the MCHergic system regulates the activity of the DR through a neurohumoral pathway (by volume conduction through the ventricular system) complementing its regulation via direct neuronal projections (Torterolo et al., 2008).

**Figure 3:**
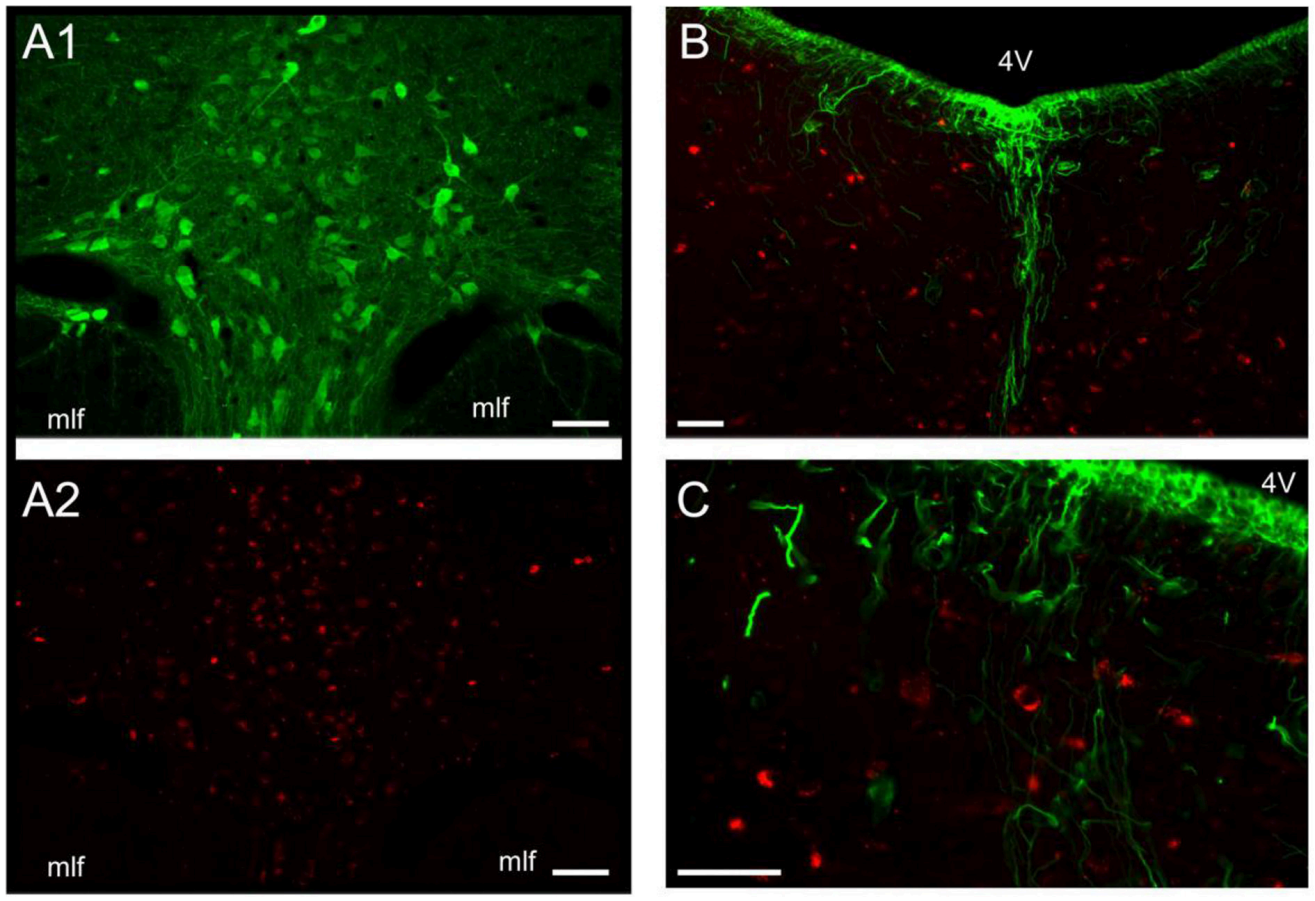
**MCH-rhodamine is internalized by DR neurons**. **(A1)** Photomicrographs of the DR of the cat illustrating serotonin immunolabeled neurons. **(A2)** DR neurons of the same field as in **(A1)** that are labeled with rhodamine (these neurons internalized MCH-rhodamine). Note that these MCH-rhodamine labeled neurons are mainly located in the same area as serotonergic neurons. The internalization of MCH strongly suggests that these neurons present MCH receptors. **(B,C)** sections were immunolabeled to detect vimentin, a marker of tanycytes in the adult cat. These photomicrographs of the DR show rhodamine fluorescence within DR neurons (red). The rhodamine-labeled neurons indicate that these neurons internalized MCH-rhodamine. Note that these neurons are located in close relationship to tanycytes (green). 4 V, fourth ventricle; mlf, medial longitudinal fascicle. Calibration bars: 100 μm. Original microphotographs taken from the data set of Devera et al. (2015).

**Figure 4:**
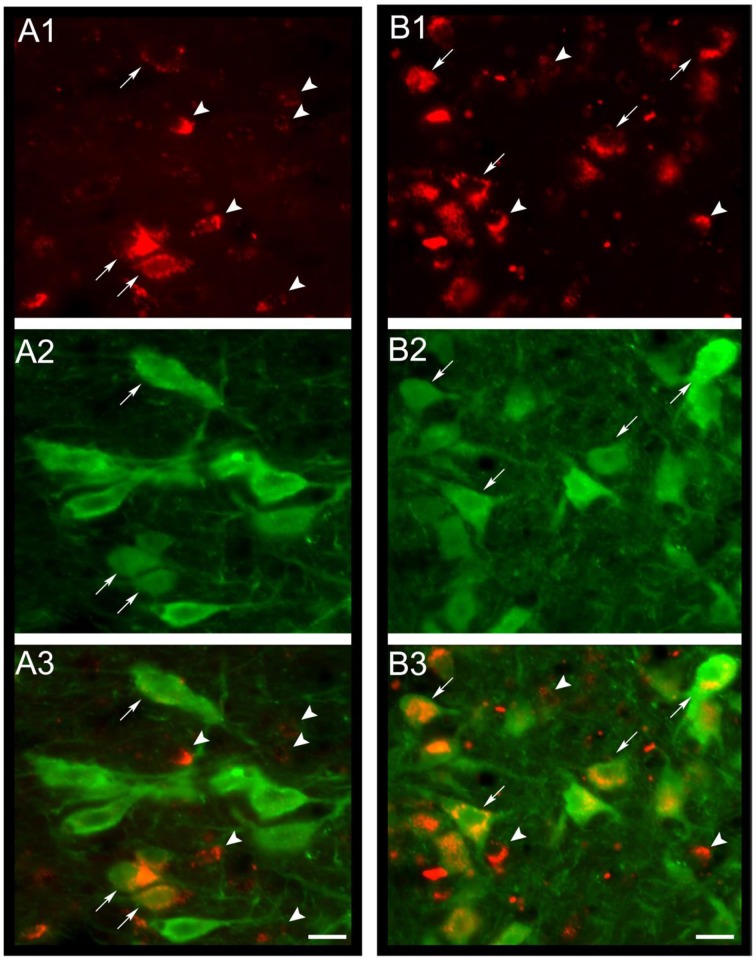
**MCH-rhodamine is internalized in DR serotonergic neurons**. In **(A1,B1)**, the photomicrographs shows that MCH-rhodamine (red) was internalized by DR neurons in the cat. Photomicrographs in **(A2,B2)** depict neurons with serotonin immunoreactivity (green). Superimposition of both photomicrographs is shown in **(A3,B3)**. It is readily observed that MCH-rhodamine is present in serotonergic (arrows) and non-serotonergic neurons (arrowheads). Calibration bars: 20 μm. Orginal microphotographs taken from the data set of Devera et al. (2015).

A wide distribution of MCHR-1 has been identified in the CNS of the rat, which coincides with the distribution of MCHergic fibers (Lembo et al., 1999; Saito et al., 2001). By a novel approach, utilizing intra-cerebro-ventricular administration of MCH labeled with a fluorescent tag (Rhodamine), Devera et al. (2015) have recently shown in cats and rats that neurons of the DR internalize MCH-rhodamine, indicating that they express receptors for MCH (Figures 3A1,A2). In the cat, there is a particularly high density of neurons with MCH receptors surrounding the basal processes of tanycytes within the DR (Devera et al., 2015; Figures 3B,C). MCHR-1 are present both in serotonergic and non-serotonergic neurons of the DR (Figure 4). Some of the non-serotonergic neurons were demonstrated to be GABAergic (Devera et al., 2015). Recently, MCHR-1 mRNA has been identified in serotonergic neurons of the ventro-medial and lateral wing areas of the DR of mice (Spaethling et al., 2014).

### MCHergic neurons are active during sleep

Using the Fos protein as a marker of neuronal activity, it has been shown that MCHergic neurons are active during REM sleep in the rat (Verret et al., 2003). Furthermore, Hassani et al. (2009) have recorded MCHergic neurons in non-anesthetized animals. These neurons have a very low frequency of discharge during wakefulness, their firing rate increases slightly during NREM sleep and reaches the maximum level of activation during REM sleep (Hassani et al., 2009). However, even during this state the average discharge rate was still quiet low (approximately 1 Hz) comparing with other neuronal groups (ibid.).

### Quantification of MCH during wakefulness and sleep

The concentration of MCH in the CSF of rats increases during the light phase, when the animals are predominantly asleep, while it decreases during the dark period when rats are mainly awake (Pelluru et al., 2013). Utilizing the *in vivo* microdialysis technique, it has been shown that the release of MCH in the amygdala of patients with treatment-resistant epilepsy is minimal during active wakefulness with social interactions, increases after eating (consummatory behavior), and reaches a maximum level at sleep onset (Blouin et al., 2013).

### Studies with genetically modified animals

Studies of preproMCH and MCHR-1 knockout mice indicate that the sleep architecture of these animals is altered. Mice lacking MCH, sleep less than wild-type animals (Willie et al., 2008). Moreover, in response to fasting, MCH deficient mice became hyperactive and show a marked decrease in REM sleep.

A study in MCHR-1 knockout mice showed an unexpected hypersomniac-like phenotype, both in basal conditions and after total sleep deprivation, compared to wild-type mice (Adamantidis et al., 2008). According to the authors, these surprising effects might be produced by compensatory mechanisms that have been identified as potential limitations of the gene-targeting approach. In contrast, Ahnaou et al. (2011) described an increase of wakefulness and a reduction of NREM sleep in MCHR-1 knockout mice, which agrees with the currently proposed role of MCH in the regulation of sleep-wake states. Moreover, restraint stress further increases wakefulness and reduces both NREM and REM sleep in these mutant mice (Ahnaou et al., 2011).

### Administration of MCH or MCHR-1 receptor antagonists

Intracerebroventricular administration of MCH in the rat produces a marked increase in REM sleep and a moderate enhancement in the time spent in NREM sleep (Verret et al., 2003). Furthermore, the systemic administration of MCHR-1 antagonists decreases both REM and NREM sleep and increases wakefulness (Ahnaou et al., 2008).

Microinjection of MCH into the DR of the rat facilitates the generation of REM sleep (Lagos et al., 2009). Conversely, the immunoneutralization of endogenous MCH within the DR (through the microinjection of anti-MCH antibodies) produces the opposite effect (Lagos et al., 2011a). Preliminary studies in cats (where the two types of MCH receptors are active) have also shown that MCH microinjections into the DR produced an increase in REM or NREM sleep depending on the exact location of the microinjection sites (Devera et al., 2007).

MCH also promotes REM sleep when microinjected into either the basal forebrain of the rat or the NPO of the cat, two areas related to the generation of this behavioral state (Torterolo et al., 2009b; Lagos et al., 2012). Noradrenergic “REM-off” neurons of the LC are critically involved in the generation of REM sleep and in the pathophysiology of MD (Itoi and Sugimoto, 2010; Brown et al., 2012). Interestingly, microinjections of MCH into this nucleus also produce a marked increase in REM sleep (Monti et al., 2015). In contrast, the administration of MCH into the ventro-lateral preoptic area (VLPO), a NREM sleep promoting area, induced NREM sleep (Benedetto et al., 2013).

### Experimental activation of MCHergic neurons induces sleep

Recent optogenetic studies have confirmed the role of MCH in sleep generation (Jego et al., 2013; Konadhode et al., 2013; Tsunematsu et al., 2014). Konadhode et al. (2013) inserted the gene for the photosensitive rhodopsine-2 cation channel in MCHergic neurons of mice, and specifically stimulated MCHergic neurons. Stimulation induced a decrease in the latency to sleep, reduced the duration of wakefulness and increased the total time spent in NREM and REM sleep during the night, whereas it increased the depth of sleep during the day (ibid.). The authors hypothesized that MCHergic neurons are able to counteract the actions of the activating systems. Consequently, it was concluded that MCHergic agonists might be useful for the treatment of insomnia.

Jego et al. (2013) found that acute optogenetic activation of MCH neurons at the onset of REM sleep extended the duration of REM sleep episodes. In contrast, their acute optogenetic silencing reduced the frequency and amplitude of the hippocampal theta rhythm during REM sleep without affecting the duration of the episodes.

Tsunematsu et al. (2014) showed that acute optogenetic activation of MCH neurons at 10 Hz induced transitions from NREM to REM sleep and increased REM sleep time. Acute optogenetic silencing of MCHergic neurons had no effect on any vigilance state. On the contrary, temporally-controlled ablation of MCH neurons by cell-specific expression of diphtheria toxin A increased wakefulness and decreased NREM sleep duration without affecting REM sleep (Jego et al., 2013; Konadhode et al., 2013; Tsunematsu et al., 2014). The authors concluded that acute activation of MCHergic neurons is sufficient, but not necessary, to trigger the transition from NREM to REM sleep and that MCHergic neurons also play a role in the initiation and maintenance of NREM sleep.

### Effect of MCH on serotonergic neurons of the dorsal raphe

**Figure 5:**
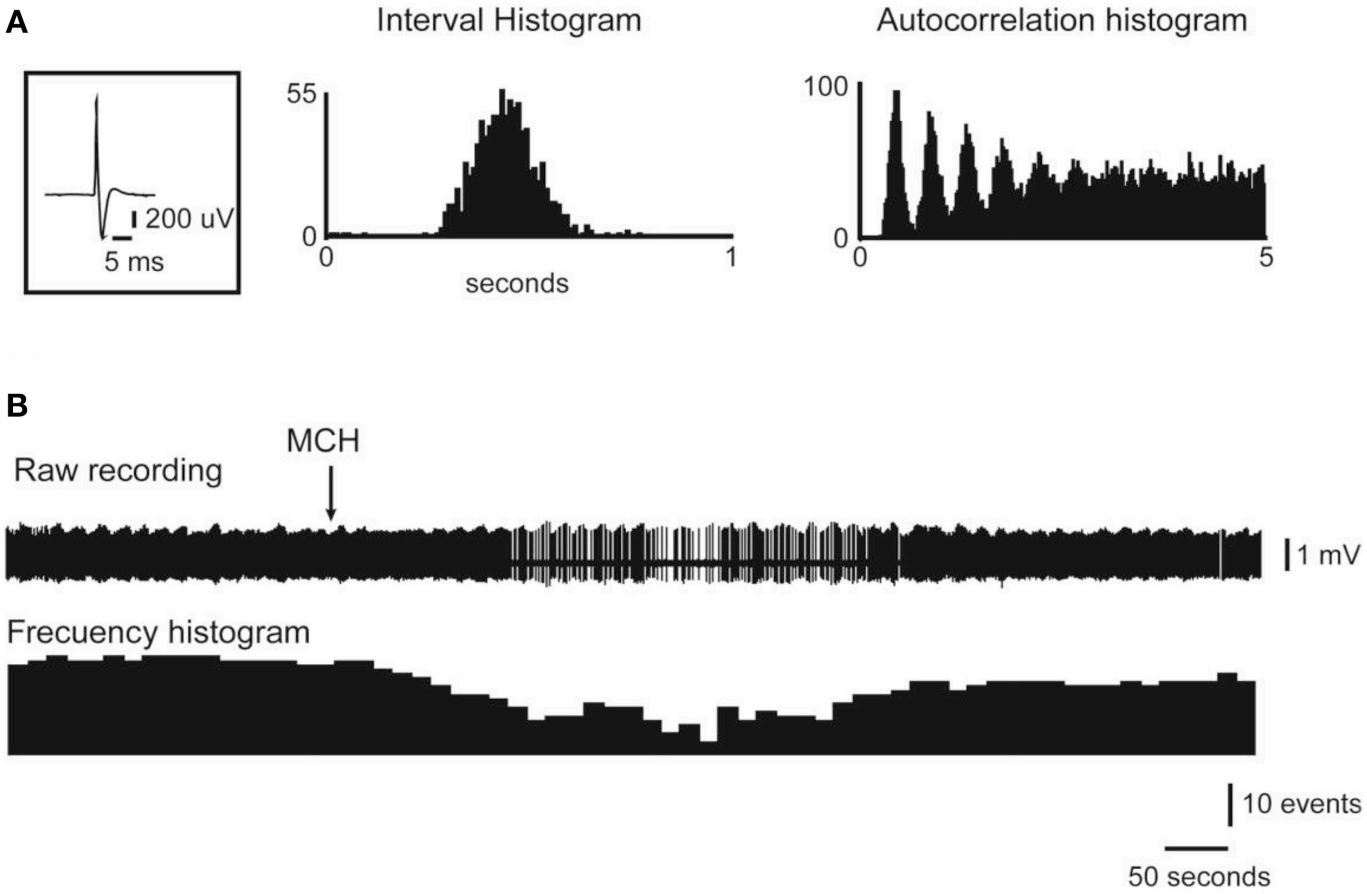
**Juxtacellular administration of MCH reduces the activity of DRN neurons**. The action potential average, the interval histogram and the autocorrelation histogram of a representative DR neuron are presented in **(A)**. The raw recording and frequency histogram are shown in **(B)**. Note that the application of MCH (indicated by the arrow) produced a decrease in the firing rate. Original Figure taken from the data set of Devera et al. (2015).

Utilizing *in vivo* extracellular recordings, we determined that the intracerebroventricular or juxtacellular application of MCH inhibits the discharge of the majority of DR neurons (Devera et al., 2015); some of these neurons were presumed to be serotonergic, according to their electrophysiological and pharmacological characteristics. Figure 5 presents an example of the inhibitory effect of the juxtacellular application of MCH on a representative DR neuron. In agreement with these electrophysiological results, *in vivo* microdialysis studies have shown that the perfusion of low concentrations (30 μM) of MCH into the DR elicited a significant decrease in extracellular serotonin levels within this region (Urbanavicius et al., 2013, 2015b).

## Role of the MCHergic system in the pathophysiology of depression

As mentioned above, the large density of MCHergic fibers in the DR, the expression of MCHR-1 in serotonergic neurons, as well as MCHergic projections toward the limbic system, suggest a relevant role of MCH in the control of emotional states. At the same time, MCH facilitates REM sleep (REM sleep is increased in MD) and stimulates the hypothalamus-pituitary-adrenal axis (which is over-activated in MD). These data also suggest that hyperactivity of the MCHergic system is related to certain aspects of MD.

There are several evidences that relate MCH and MD (see below); however, most of the data are from preclinical studies. Hence, it is important to be cautious, because the results from animal models of depression may not correspond with clinical findings (recently reviewed by Belzung, 2014).

Borowsky et al. (2002) demonstrated that the MCHR-1 antagonist, SNAP-7941, possesses antidepressant and anxiolytic effects in animal models of MD (Borowsky et al., 2002). Similar results have been presented by other authors (Shimazaki et al., 2006; David et al., 2007; Chung et al., 2010). In addition, following a 5-week exposure to repeated chronic mild stress (an ethologically relevant animal model of depression), in C57Bl/6J mice there is an increase in the hippocampal gene expression of MCHR-1. This increased gene expression was reversed by chronic fluoxetine treatment (Roy et al., 2007).

The importance of the MCHergic system in MD is emphasized in a recent study which suggests that an increase in the expression of preproMCH and consequent MCH receptor down-regulation could be a biomarker of the severity of depressive disorders (García-Fuster et al., 2012).

Because MCHergic neurons regulate energy homeostasis, it is expected that this function would be altered during MD; in fact, changes in body weight are characteristic of patients with MD (American-Psychiatric-Association, 2013). At the same time, preclinical studies have demonstrated that MCHR-1 antagonists are not only antidepressants, but also have strong anti-obesity effect (Shimazaki et al., 2006; Chung et al., 2010).

As mentioned before, MCH is expressed in neurons of the medial preoptic nucleus of the female rat, which is a critical area in the control of maternal behavior, but only during the post-partum period (Rondini et al., 2010). Recently, Benedetto et al. (2014) have shown that microinjections of MCH into this preoptic nucleus decrease active maternal behaviors during the early post-partum period. Hence, it would be important to know whether a dysfunction of MCH-containing neurons, which are exclusively present during the post-partum period is related to emotional imbalances that take place in 75–80% of mothers between 3 and 5 days after delivery (Lee, 1998).

### MCH and raphe nuclei: role in depression

In Table 2, we summarized the effects of MCH agents applied into the DR and MR. In direct relation with MD, we explored the role of MCH within the rat DR in the generation of depressive-like behaviors (Lagos et al., 2011b; Urbanavicius et al., 2015a). MCH microinjections into the DR induce a pro-depressive response evaluated in the FST. MCH produced a significant increase in immobility time without affecting locomotor activity; this response is opposite to the prototypical antidepressant effect. The response was blocked when the animals were pretreated systemically with fluoxetine or nortriptyline, which are selective serotonin and noradrenergic reuptake inhibitors antidepressant, respectively (Lagos et al., 2011b; Urbanavicius et al., 2015a). Furthermore, the pro-depressive effect was also suppressed by the intra-DR microinjection of ATC-0175, a selective MCHR-1 antagonist. Additionally, immunoneutralization of endogenous MCH produced an antidepressant effect, since a significant reduction of immobility time was observed (Lagos et al., 2011b). This response was accompanied by an increase in the swim time; this effect is associated with an increase in serotonergic neurotransmission (Lagos et al., 2011b).

**Table 2:** **MCH and the raphe nuclei**.

**Substance**	**Site**	**Strategies**	**Main effect**	**References**
MCH	DR	Microinjection and sleep recording	Increases REM sleep. Moderate increases in NREM sleep	Lagos et al., 2009
Anti-MCH antibody	DR	Microinjection and sleep recording	Decreases REM sleep. Increases wakefulness	Lagos et al., 2011a
MCH	DR	Microinjection, FST	Increases immobility time. This effect is blocked by systemic administration of fluoxetine and nortriptyline	Lagos et al., 2011b; Urbanavicius et al., 2015a
Anti-MCH antibody	DR	Microinjection, FST	Decreases immobility time	Lagos et al., 2011b
MCHR1 antagonist (ATC0175)	DR	Microinjection, FST	Reverts the pro-depressive effect of microinjections of MCH into the DR	Urbanavicius et al., 2015a
MCH	MR	Microinjection, FST	Increases immobility time	López Hill et al., 2013
MCH	DR	Unit recording, intraventricular and juxtacellular administration of MCH	Inhibit serotonergic and non-serotonergic neurons	Devera et al., 2015
MCH	DR	Microdialysis of serotonin, local perfusion of MCH	At low doses decreases serotonin release	Urbanavicius et al., 2013, 2015b

*All the experiments were performed in rats. FST, forced swimming test*.

Considering the electrophysiological and *in vivo* microdialysis results described above, the pro-depressive effect induced by MCH could be generated by the inhibition of DR serotonergic activity elicited by this neuropeptide.

Recent studies have also shown that the MR is also involved in the pro-depressive effect induced by MCH (López Hill et al., 2013). Serotonergic neurons of the MR express are also inhibited by the intracerebroventricular or juxtacellular administration of MCH (Pascovich et al., 2011).

In this regard, Roy et al. (2006) demonstrated that the chronic deletion of MCHR-1 have altered serotonergic neurotransmission in the prefrontal cortex, one of the main target structures of the serotonergic system and highly associated with the control of emotional processes (Roy et al., 2006). Of note, other areas such as the nucleus accumbens and the basolateral amygdala, have been also proposed to be involved in the pro-depressive effect of MCH (Georgescu et al., 2005; Kim et al., 2015).

Our working hypothesis is that MD is associated with an increase in the activity of MCHergic neurons. In accord with this hypothesis, the antagonism of MCH would be effective in treating MD (Shimazaki et al., 2006; Chung et al., 2010). If our hypothesis is correct, we would expect that antidepressants would decrease the activity of these neurons. Interestingly, it has been observed that the acute treatment with escitalopram (an antidepressant of the SSRI group) inhibits REM sleep rebound that follows a sleep deprivation protocol and promotes a reduction in the activity of MCHergic neurons (Katái et al., 2013). Both set of data support our hypothesis. In addition, in electrophysiological recordings of identified MCHergic neurons *in vivo*, we observed that the juxtacellular application of fluoxetine decreases MCHergic neuronal activity (Pascovich et al., 2014). Interestingly, in accordance with our results, Kim et al. (2015) showed that the antidepressant effect of exercise is associated with the suppression of MCHergic activity within the basolateral amygdala (Kim et al., 2015).

## Conclusions and future directions

Preclinical studies suggest that the MCHergic system is involved in the control of REM sleep and depression. The role of MCHergic system in the regulation of sleep, especially REM sleep is well-established. In fact, Luppi et al. (2013) introduced a new model of REM sleep in which the MCHergic neurons plays a REM sleep promoting effect. However, the electrophysiological effect of MCH on the mesopontine areas critical for REM sleep generation (according to this model), such as the ventro-lateral periaqueductal gray and the sublaterodorsal nucleus are still to be tested. Another important issue in the research agenda is to know the role of the other neurotransmitters and neuromodulators (such as neuropeptide E-I, neuropeptide G-E, GABA) that are co-localized with MCH (Macneil, 2013).

Our working hypothesis is that an abnormal increase in the activity of MCHergic neurons is involved in the pathophysiology of depression. Hence, it would be important to determine whether depressive patients have higher levels of MCH in the CSF as compared to normal subjects. In this respect, Schmidt et al. (2015) have shown that MCH serum levels decrease in major depressive disorder following 4 weeks of antidepressant treatment (Schmidt et al., 2015). However, measurements of MCH within the CSF are of rule.

Finally, preclinical studies have demonstrated that MCHR-1 antagonists have an enormous potential as antidepressant drugs (Shimazaki et al., 2006; Chung et al., 2010). Their efficacy, rapid start of action as well as apparent absence of severe adverse events tends to support the proposal.

### Conflict of interest statement

The authors declare that the research was conducted in the absence of any commercial or financial relationships that could be construed as a potential conflict of interest.
